# Autoantibodies in Rheumatoid Arthritis: Historical Background and Novel Findings

**DOI:** 10.1007/s12016-021-08890-1

**Published:** 2021-09-08

**Authors:** Maria V. Sokolova, Georg Schett, Ulrike Steffen

**Affiliations:** 1 Department of Internal Medicine 3 - Rheumatology and Immunology, Friedrich-Alexander-Universität Erlangen-Nürnberg and Universitätsklinikum Erlangen, Universitätstrasse 25a, 91054 Erlangen, Germany; 2 Deutsches Zentrum Für Immuntherapie, Friedrich-Alexander-Universität Erlangen-Nürnberg and Universitätsklinikum Erlangen, Erlangen, Germany

**Keywords:** Rheumatoid arthritis, ACPA, Autoimmunity, Immunoglobulin

## Abstract

Autoantibodies represent a hallmark of rheumatoid arthritis (RA), with the rheumatoid factor (RF) and antibodies against citrullinated proteins (ACPA) being the most acknowledged ones. RA patients who are positive for RF and/or ACPA (“seropositive”) in general display a different etiology and disease course compared to so-called “seronegative” patients. Still, the seronegative patient population is very heterogeneous and not well characterized. Due to the identification of new autoantibodies and advancements in the diagnosis of rheumatic diseases in the last years, the group of seronegative patients is constantly shrinking. Aside from antibodies towards various post-translational modifications, recent studies describe autoantibodies targeting some native proteins, further broadening the spectrum of recognized antigens. Next to the detection of new autoantibody groups, much research has been done to answer the question if and how autoantibodies contribute to the pathogenesis of RA. Since autoantibodies can be detected years prior to RA onset, it is a matter of debate whether their presence alone is sufficient to trigger the disease. Nevertheless, there is gathering evidence of direct autoantibody effector functions, such as stimulation of osteoclastogenesis and synovial fibroblast migration in in vitro experiments. In addition, autoantibody positive patients display a worse clinical course and stronger radiographic progression. In this review, we discuss current findings regarding different autoantibody types, the underlying disease-driving mechanisms, the role of Fab and Fc glycosylation and clinical implications.

## Introduction

Rheumatoid arthritis (RA) is a chronic inflammatory joint disease, which is considered to be of predominantly autoimmune origin. The disease was first described in 1800 by Dr. Landré-Beauvais and was initially regarded as a form of gout. Only in the mid-nineteenth century was it distinguished from actual gout based on the uric acid measurement [1, 2]. RA is characterized by a progressive course, which in the absence of treatment is ultimately leading to pannus formation and joint destruction. Since its first description, the understanding of RA pathophysiology has evolved immensely. As is the case with most rheumatic diseases, the pathogenesis of RA is very complex and influenced by genetic and environmental factors, microbiota, barrier layers, and hormones. The main event in the pathogenesis is still considered to be the breach of tolerance followed by one or multiple “second hits” [3].

Although autoantibodies in RA are a topic of extensive research, the understanding of their precise role in RA development is still far from being complete. In the last decades, many self-antibodies have been identified as hallmarks of RA, starting with rheumatoid factor (RF) first described in 1957 in the sera of RA patients [4]. Although RF has proven to be not specific enough for RA and is present in a variety of other conditions, its discovery was the first step towards recognition of the important role played by autoimmunity in the pathogenesis of arthritis. In 1964 the so-called antiperinuclear factor was described in the serum of patients with RA and several other diseases, including systemic lupus erythematosus and ankylosing spondylitis [5, 6]. The nature of these antibodies was not perceived, and until 1978, when keratin-specific antibodies were described, there were no major discoveries in the field of autoantibodies in RA. Finally, in the 1990s, the crucial understanding of citrullination and its importance for autoimmunity in RA was acknowledged [7]. The success of B-cell-depleting agents in RA, which was first observed with rituximab approved for the treatment of RA in 2007, further confirmed the driving role of adaptive immunity in the pathogenesis of the disease.

Despite the generally accepted concept of RA as a disease of autoimmune origin, in the regular clinical practice, there is a significant proportion of patients (up to 40% [8]) classified as “seronegative RA” — assuming they do not possess autoantibodies. Seronegativity is usually defined by the absence of anti-citrullinated protein antibodies (ACPA) and/or IgM-RF. However, research in recent years has also shown that the presence of fine-specificities [9] or newly discovered autoantibodies [10], as well as re-diagnosis to other rheumatic diseases [11] are making this group highly heterogenic and its place in the classification of musculoskeletal diseases remains to be clarified.

The autoantibody response in RA is an intensively researched field of high importance and interest. In this review, we discuss the latest findings concerning autoantibodies in RA, their clinical significance and the current consensus on the place of autoantibodies in the pathogenetic cascade.

## Various Autoantibodies in Patients with RA

Sensitivity and specificity of various autoantibodies identified in RA are displayed in Table 1.

**Table 1:** Sensitivity and specificity of various autoantibodies in rheumatoid arthritis

Autoantibody	Sensitivity/specificity	References
**RF**	60–90%/85%	[145, 146, 52]
**ACPA (CCP2)**	41–74.8%/91–100%	[5, 13, 146, 45]
**ACPA (CCP3)**	77.5–78.8%/87.8–96.6%	[5, 13]
**ACPA (CCP3.1)**	74–83%/89.6–98.3%	[5, 13]
Anti-CarP (^*^carbamylated fetal calf serum)	35–46.8%/91.95–92.8%	[146, 147]
**Anti-acetylated vimentin antibodies**	36.6%/86.2%	[45]
**Anti-PAD4-antibodies**	21.72–40%/90.36–98.8%	[65, 148, 10]
**Anti-PTX3**	25.52%/91.37%*	[10]
**Anti-DUSP11**	27.93%/90.36%	[10]

*RF* rheumatoid factor, *ACPA* anti-citrullinated protein antibodies, *CCP* cyclic citrullinated peptide, *Anti-CarP* anti-carbamylated protein antibodies, *DUSP11* dual specificity phosphatase 11, *PAD* peptidyl arginine deiminase, *PTX3* pentraxin 3

^*^Specificity should be regarded with caution, since these antibodies have been identified in other rheumatic conditions, such as systemic lupus erythematosus or anti-neutrophil cytoplasmic antibody-vasculitis [53, 54]

### ACPA

The concept of ACPA was born when citrullinated epitopes were discovered to be “the common ground” of recognition by both the “perinuclear factor” and anti-keratin antibodies. It was noticed that these antibodies both reacted with fillagrin but not with pro-fillagrin, and it was hypothesized that a posttranslational modification could take place [7, 12]. Schellekens et al. also noticed already at that time that ACPA could be detected very early in the disease course [12]. Since then, extensive research has been conducted on the role and diagnostic importance of ACPA. ACPA display high sensitivity (60–78%) and specificity (86–99%) for RA [5, 13]. Today, various ACPA tests are available (reviewed in detail in [5]). Currently, the most widely used test for the measurement of ACPA is the cyclic citrullinated peptide (CCP) 2 test, first introduced in 2000. In the CCP3 test, additional citrullinated epitopes are introduced to the same protein structure. Both tests are similar in their performance [5] and the additional advantage of the CCP3 test (which is also more expensive than the standard CCP2) is not completely apparent. However, CCP3.1 showed a slightly higher specificity compared to CCP2 and anti-modified citrullinated vimentin (MCV) tests [13], and CCP3-positivity was shown to add predictive value to the CCP2 test for the development of RA in individuals-at-risk [14]. The anti-MCV antibody test is also universally used and has comparable sensitivity and specificity profiles to CCP tests.

In the process of citrullination, peptidyl arginine is being transformed to citrulline by the calcium-dependent enzyme peptidylarginine deiminase (PAD). There are multiple forms of PADs, which differ in their localization and function. PAD2 and PAD4 play a prominent role in RA not only due to direct production of citrullinated antigens but also by contributing to neutrophil extracellular trap (NET) formation via citrullination of nuclear proteins [15, 16].

ACPA recognize citrullinated parts of various targets and are therefore somewhat “promiscuous” in their recognition profile, binding to proteins like vimentin, α-enolase, type II collagen (CII), fibrinogen, fillagrin, fibronectin, immunoglobulin-binding protein (BiP), and potentially various others, provided they contain accessible citrullinated epitopes [5, 17–21]. Consequently, oftentimes these antibodies are showing extreme cross-reactivity. In case of collagen II (CII), it seems that citrullination is crucial for the recognition of epitopes by autoantibodies in humans, in contrast to mouse antibodies in the collagen-induced arthritis model, where citrullination prevents the binding to CII [22, 23].

New epitopes for the recognition by ACPA are being constantly discovered and antigen arrays are getting more attention as a means to investigate the complete “citrullinome” and to identify other potential targets [10, 24, 25]. It has been demonstrated in the last years that glycine motifs next to the cit-position are essential for ACPA binding [24, 26–28]. Hydrogen bonds are responsible for the interaction of complementarity-determining regions with the antigen main chain and ACPA show limited interaction with protein side chains. Both factors are considered to be the basis for their wide cross-reactivity [27]. However, a specific three-dimensional fold structure of proteins also interferes with binding, and the cross-reactivity has therefore its limits.

The cross-reactivity profile is not uniform and can differ between ACPA, as was shown for monoclonal antibodies and polyclonal ACPA from the serum of RA patients [26, 27, 29, 30]. The concept of “promiscuous” and “private” ACPA was suggested recently based on studies demonstrating the presence of more selective (“private”) antibodies alongside classical cross-reactive (“promiscuous”) ACPA [19]. It has been shown for instance that some ACPA recognize not both, but only either one of citrullinated residues in CII. Interestingly, only non-cross-reactive antibodies were shown to bind to cartilage proteins [22].

### AMPA

In the recent years, autoantibodies targeting various other posttranslational modifications have been discovered. While citrulline is a product of enzymatic conversion of arginine, other modifications happen mostly to lysine residues. This amino acid can be transformed non-enzymatically into homocitrulline via addition of isocyanic groups (process known as carbamylation) or into malondialdehyde (MDA)-acetaldehyde (MAA)-lysine. MDA is a product of lipid peroxidation by reactive oxygen species and can be attached to a variety of aminoacidic residues, such as glutamine, asparagine, arginine, and histidine [31]. Another frequent enzymatic modification of lysine is acetylation. It has been demonstrated that ACPA are cross-reactive not only towards various citrullinated peptides, but also towards other posttranslational modifications [28, 32–35]. Therefore, the concept of anti-modified protein antibodies (AMPA) was suggested. AMPA are extensively reviewed elsewhere [34, 36]; hence, here we focus only on the milestones and most recent findings.

It is worth mentioning that AMPA are present mostly in ACPA-positive patients, suggesting a cross-reactive nature. Despite that, the recognition profile of anti-carbamylated protein antibodies (anti-CarP) for example only partially overlaps with that of ACPA [32–34, 37, 38]. It is noteworthy that homocitrulline, the product of carbamylation, highly resembles citrulline and only contains one additional CH2 group, which might be partially responsible for the cross-reactivity. Another reason is suggested to be the Cit-Gly “consensus motif” [26, 39]. Interestingly, isocyanic acid for the process of carbamylation can be generated by myeloperoxidase from thiocyanate, which is abundant in the blood of smokers [40]. This might provide an additional link between the risk of RA and smoking. Importantly, anti-CarP antibodies were found to be associated with interstitial lung disease (ILD) in RA patients even after adjusting for other factors, including smoking; though anti-CarP were more prevalent among smokers [41].

Similarly, anti-MAA antibodies are present mostly in ACPA-positive patients as well. The expression of MAA-modified-proteins is found in the synovium of RA patients, but not in osteoarthritis (OA) patients, and co-localizes with citrullinated proteins [31, 42]. However, the data on MAA cross-reactivity with citrullinated proteins are controversial. In another study, monoclonal anti-MAA antibodies did not cross-react with either CCP, carbamylated, or 4-hydroxynonenal (HNE)-carbonylated proteins [43]. Interestingly, just like ACPA, anti-MAA can bind various peptides containing MAA modification. Similarly to carbamylation, MAA overexpression was identified in the lung tissue of RA patients with ILD, and anti-MAA antibodies were associated with ILD in these patients [44].

Anti-acetylated protein antibodies are mostly reserved to ACPA-positive patients as well [45, 46]. Anti-acetylated ornithine antibodies slightly improved the specificity of EULAR/ACR 2010 diagnostic criteria in one study, but not in case of seronegative (ACPA- and RF-negative) RA [46]. By contrast, the addition of anti-CarP antibodies to the classification criteria was favoring sensitivity, while the specificity was reduced; although both effects were very minor and not cost-effective [47].

There is still lack in comprehensive understanding of how AMPA responses develop. Recent studies also show that the process is most likely to be highly individual and heterogeneous, with various monoclonal ACPA showing different specificity profiles and exhibiting distinct effector functions [28, 39].

### RF

The discovery of RF served as a first evidence of autoimmunity in RA. It encompasses immunoglobulins of IgM, IgG, and IgA classes targeting the Fc-fragment of IgG. However, RF can be present in a variety of other rheumatologic and non-rheumatologic conditions, including a broad spectrum of infectious diseases — most importantly hepatitis C-virus infection and cryoglobulinemia [48], as well as cancer [49–52]. The specificity of RF in RA is estimated to be 85% at best, and the sensitivity to be in the range between 60 and 90% [52]. Compared to ACPA, RF is slightly more sensitive; however, it certainly lacks specificity. Still, both tests are included in the current EULAR/ACR diagnostic criteria. As will be mentioned later in one of the sections, RF of IgA class is probably playing a more significant role compared to other immunoglobulin classes.

### Antibodies to Native Proteins

Recently, several novel candidates unrelated to posttranslational modifications were suggested as promising markers in RA. The concept of seronegative RA is problematic, both from diagnostic and pathogenetic perspectives. Some novel candidate autoantibodies present in this subset of patients have been suggested, including anti-pentraxin 3 (PTX3) and anti-dual specificity phosphatase 11 (DUSP11) antibodies. These antibodies were present in about 30–40% of both ACPA-positive and ACPA-negative patients in one study [10]. In the same study, additional candidates were suggested. However, these novel antibodies are lacking sensitivity (which was below 30%). Moreover, in another study, anti-pentraxin-related protein 3 (PTX3) antibodies were found in about 50% of systemic lupus erythematosus patients and in none of RA patients [53]. These antibodies have also been reported in anti-neutrophil cytoplasmic antibody (ANCA)-associated vasculitis (about 40% of patients in one cohort were positive) [54]. Therefore, the data on the specificity of these antibodies for RA need to be considered with caution.

In another array-based study, antibodies against a total of 102 native proteins (76 in ACPA-negative RA patients and 86 recognized by the serum of ACPA-positive patients) were found to be specific for RA [55]. Some of the antigens were found in synovial fluid [56], but this still needs to be validated in other studies.

The better known and studied anti-PAD-antibodies are slightly more sensitive (20–70%) and specific (77–97%) for RA compared to other antibodies against native proteins [15]. These antibodies are mostly associated with ACPA but can also be identified in up to 19% of ACPA-negative RA patients [15].

Considering all this, it seems that signs of autoimmunity can be found in at least a subset of “seronegative” patients. The autoantibody spectrum might be broader than we currently know and is likely not reserved to citrullinated proteins. However, it is hard to implicate these findings into the daily clinical practice, since the benefit of testing for other autoantibodies remains to be proven.

## Factors Contributing to Autoantibody Production in RA

The presence of ACPA is highly associated with the human leukocyte antigen (HLA)-shared epitope (SE) — the susceptibility loci encoding a particular amino acid sequence in the third hypervariable region in the HLA-DR beta-chain 1 (HLA-DR4, HLA-DR1, or HLA-DR10) [57, 58]. HLA shared epitope is linked to both the risk of developing RA and the risk of having ACPA-positive RA, but not to the risk of having ACPA alone [59], which indicates its role in both ACPA production and the effector phase of arthritis. There also seems to be a ‘gene-dose’-dependent effect, with patients harboring two alleles or HLA-DRB1.04 having higher ACPA titers [57, 58].

Since the role of HLA is to present antigens to T cells and to aid B cells in getting T cell-help, it was logically proposed that SE alleles are contributing to the risk of having ACPA and arthritis due to a higher ability of the MHC to bind citrullinated epitopes. However, this hypothesis, though very plausible, has its flaws. While the higher binding affinity of citrullinated vimentin as opposed to its native form has been shown for three HLA-SE alleles (HLA-DRB1*01:01, *04:01, and *04:04) [60, 61], at the same time, citrullination of fibrinogen was demonstrated to have no influence on its binding to HLA, and there were no differences in T cell proliferation upon stimulation with citrullinated or non-citrullinated proteins in HLA-DRB1* patients [62]. Overall, the data on this topic are controversial [57]. Moreover, a novel hypothesis, which has been supported by some recent studies, has emerged. It is suggested that the HLA-DR alleles might contribute to preferable binding of PAD4 peptides, and not citrullinated or native fibrinogen [63, 64]. The authors propose an intriguing hypothesis that PAD is serving as a hapten for citrullinated proteins, evoking T cell immune response and triggering the production of ACPA. This is, however, somewhat challenged by the evidence of ACPA response usually preceding the anti-PAD-antibody response [65].

Additional susceptibility gene variants for ACPA-positivity, such as in PTPN22 [66] and in HLA-DQ and HLA-DP [67] have been described.

Other well-known risk factors for ACPA-positivity are environmental stressors. Smoking is estimated to dose-dependently contribute to at least 35% of ACPA-positive RA cases and is associated synergistically with the HLA-DRB1 shared epitope [68, 69]. In a large cohort of ACPA-positive individuals at-risk and ACPA-positive RA patients, smoking has been found to be associated with both ACPA- and anti-carbamylated protein antibody (anti-CarP)-positivity [70]. Interestingly, the presence of secretory IgA ACPA in serum of RA patients was found to be associated with smoking [71]. Smoking is considered to contribute to ACPA production via excessive citrullination in lung tissue. This is backed up by immunohistochemistry showing citrullinated proteins and increased PAD expression in the bronchoalveolar lavage from smokers [67, 72]. If an individual in addition has one or more risk-alleles, which provide the needed T-cell help, this combination is considered to increase the risk of RA up to 21-fold [73].

Periodontitis is another suggested risk factor for autoantibody production in RA and individuals at-risk. First, the incidence of RA was found to be higher among patients referred for the treatment of periodontal disease than in the general population [74]. Periodontitis was more frequently found among RA patients in some studies [75, 76] and meta-analysis [77], while equal prevalence among RA and non-RA individuals was shown in others [77, 78]. These associations, if found, were also correlating with ACPA-positivity. Likewise, ACPA-positivity was found to be associated with the periodontal health in first-degree relatives of RA patients, with a higher prevalence of periodontitis if ACPA-positive [79]. Higher titers of anti-*Porphyromonas gingivalis* antibodies were also found in ACPA- or RF-positive subjects at-risk for RA [80]. *P. gingivalis* produces PAD, which could account for its possible contribution to citrullination and subsequent autoantibody production in the context of RA [77, 78]. Though data on the topic are controversial, mostly, the results point towards a possible role of periodontal disease in autoantibody production. This topic is discussed in more detail in other reviews, like [81].

Finally, the so-called leukotoxic hypercitrullination via the pore-forming leukotoxin A (LtxA) has been described for neutrophils in *Aggregatibacter actinomycetemcomitans* (Aa) infection. Antibodies to both Aa and LtxA were present in patients with RA and periodontitis [82]. Recently, the presence of anti-Aa- and anti-LtxA-antibodies was found to be associated with the measures of atherosclerosis in patients with RA in one study, suggesting periodontal disease prevention to be important, though these findings do not allow to conclude if these associations are specific for RA, since the study did not include a non-RA control group [83].

## Pathogenetic Role of Autoantibodies in RA

As ACPA are the main autoantibodies in RA, there has been interest in identifying their target antigens in the RA synovium. One study found a 1.4-fold increase in the proportion of ACPA in total IgG, IgA, and IgM in the RA synovial fluid compared to serum [84] and a 7.5-fold increase of IgG in synovial lining compared to serum [85]. Interestingly, immunohistological staining with citrulline-specific antibodies, including CCP1-specific antibodies isolated from RA serum, showed the presence of citrullinated proteins not only in RA synovium, but also in the inflamed synovium from patients with other inflammatory joint diseases [84]. Other studies identified citrullinated proteins in a wide spectrum of inflamed tissues, but only in a minority of healthy specimens, confirming the notion that citrullination is the result of an ongoing inflammation [86, 87]. Synovial tissue is infiltrated by neutrophils that might serve as major contributors to citrullination through the release of PAD4 [88]. In a more recent study, authors found higher levels of citrulline and homocitrulline in the synovial tissues of seropositive RA patients than in seronegative RA and OA (citrulline = 0,019 µg/mg in seropositive RA versus 0,007 µg/ml in both OA and seronegative RA) [87]. In the same study, the majority of citrullinated and homocitrullinated proteins were recognized by a synthetic antibody in the area of necrotic tissue in the synovium and rheumatic nodules, as well as in the synovial lining and endothelium. The expression of PAD2, PAD3, and PAD4 could be localized in the synovial lining and the expression of myeloperoxidase was identified around necrotic areas [87, 89].

The notion that ACPA could have a direct pathogenetic role has its back up in the clinical setting. ACPA-positivity is associated with bone loss in individuals at-risk for RA [90] and in the early disease [72, 91]. Overall, seropositive RA is characterized by a more aggressive inflammation and destruction compared to seronegative RA [72, 87, 92, 93], though one study showed contrasting results [94]. The presence of autoantibodies has been associated with bone loss and erosion formation (radiographic progression) [90, 95–97]. Interestingly, newborns of women with RA, although having no long-term developmental consequences, were shown to be smaller for their gestational age [98]. On the other hand, not every ACPA-positive individual progresses to RA even after several years. In a study by Hensvold et al., only 8.5% (21 of 247) of ACPA-positive individuals from a large prospective twin cohort developed RA in 3 years of follow-up [99]. In some RA patients, ACPA can be detected up to 10 years prior to diagnosis [100]. These observations challenge the concept of the pathogenicity of autoantibodies in RA.

Still, there is gathering evidence that ACPA might be exhibiting direct effector functions in RA. Studies have demonstrated ACPA to stimulate bone resorption through enhanced osteoclast differentiation, while in addition inducing TNF-α production by osteoclast precursors [101]. In a recent study, the presence of ACPA was associated with the elevation of osteoclast activation and bone resorption markers like tartrate-resistant acid phosphatase 5b (TRAP5), cathepsin K and C-terminal telopeptide of type I collagen (CTX-I) in the bone marrow of RA patients [102].

ACPA were also shown to stimulate cytokine production, specifically of TNF-α by macrophages via Fcγ receptors in vitro, and this effect was amplified by the presence of IgM RF through its conjugation with IgG [103]. Recently, Dong et al. showed that ACPA are able to induce the production of interleukin (IL)-1β by macrophages in vitro via NLRP3 inflammasome-activation through CD147, integrin β1 and Akt/NFkB signaling [104].

In another recent study, ACPA have been shown to promote migration of fibroblast-like synoviocytes (FLS) under starving conditions and upon inflammatory stimuli, such as IL-8 [105]. The effects of ACPA on FLS were elicited via their binding to citrullinated residues and were dependent on the expression of PAD-2 and PAD-4 in FLS.

A potential direct involvement of ACPA in joint pain pattern is controversially discussed. It has been demonstrated with monoclonal ACPA; however, there was a following correction, since the monoclonal antibodies used in the study were proven to be unspecific. Still, those antibodies were also showing effector functions, which raises another interesting question of whether the properties of autoantibodies are dependent on their specificity profile or are exhibited through Fc-receptors, irrespective of the Fab-recognition [106]. ACPA-positivity was not associated with pain at the time of diagnosis in a Swedish cohort of newly diagnosed RA patients [72]. On the other hand, antibodies targeting cartilage proteins (collagen II and cartilage oligomeric matrix protein) were shown to induce pain in mice before the onset of inflammation in a neuronal Fcγ-receptor-dependent manner [107].

There is limited evidence on whether mice spontaneously develop ACPA upon the induction of experimental arthritis. This might be strain-dependent [108–110]. In addition, ACPA alone are usually not sufficient to induce arthritis in mice [22, 110]. However, upon immunization with citrullinated collagen II without adjuvant, mice developed ACPA, which was associated with increased bone resorption and cartilage loss [109]. Additionally, pre-immunization with citrullinated antigen BiP has been shown to exacerbate experimental arthritis [21].

## Influence of Glycosylation on the Properties of Autoantibodies in RA

Glycosylation has been shown to considerably modulate effector functions of immunoglobulins. Particularly important is the presence of sialic acid at the glycan attached to asparagine at the position 297 — known as sialylation. It has been shown to skew the functional profile of the antibodies towards anti-inflammatory [111]. Desialylated immune complexes were shown to be potent inducers of osteoclastogenesis and treatment with the sialic acid precursor N-acetylmannosamine (ManNAc) significantly reduced the susceptibility of mice to CIA [112, 113]. It was also observed that patients with less sialylated IgG have decreased bone volume. However, in the context of strong inflammation, the role of glycosylation in the osteoclastic activity was much less significant than under physiological conditions [113].

It has been shown that peripheral blood plasmablasts of ACPA-positive RA patients have much less sialic acid residues on their surface compared to plasmablasts from healthy individuals. The same was observed for IgG [114]. Moreover, it has been demonstrated that IgG from RA patients acts less inflammatory and its pro-inflammatory potential could be boosted upon pre-treatment with the sialic acid cleaving enzyme neuraminidase. Importantly, as was shown in mouse models, the expression of the sialylating enzyme ST6GAL1 in antibody-secreting cells was impaired by cytokines from the IL-23-axis and this effect was reserved to autoantibodies [114]. In line with this study, the ACPA glycosylation profile has been shown to differ from that of total IgG in serum, as well as in synovial fluid [115].

Another important feature of ACPA is their Fab-glycosylation. This non-conserved glycosylation has been shown to negatively influence the avidity profile of ACPA and seems to be the result of the introduction of new N-glycosylation sites during the extensive somatic hypermutation process [116, 117]. In a recent study, the importance of Fab-glycosylation for the recognition of NET-antigens and citrullinated histones was demonstrated [118]. Interestingly, IgM ACPA do not appear to have this characteristic Fab-glycosylation [119]. Most recently, Fab-glycosylation was proposed to play a role in the affinity maturation and clonal selection of ACPA [120]. Interestingly, ACPA of lower avidity profile were associated with more advanced joint damage [121]. Common features of ACPA, such as their cross-reactivity, recognition of post-translational modifications and relatively low avidity (Kd predominantly in the micromolar range), are contradicting the evidence of extensive somatic hypermutations. The conflict between the extensive process of selection and the low-affinity output raises the question of how ACPA-expressing B cells avoid the survival requirement for higher affinity. Vergroesen et al. proposed that the favoring of N-glycosylation sites is required for the survival of autoreactive B cell clones. This notion is supported by their discovery that the number of N-glycosylation sites in ACPA-reactive B cells did not correlate with mutation frequency and hence is unlikely to be merely the consequence of the high ACPA mutation rate [120].

## Clinical Relevance of Autoantibodies in RA

The prevalence of ACPA in the general population is estimated to be around 1% and is increased with older age, in smokers and individuals with joint complaints [122].

Multiple autoantibodies, including ACPA, anti-PAD- and anti-CarP-antibodies, precede the onset of RA [65, 123, 124], with ACPA still rendering the highest risk [125] and appearing before clinically apparent synovitis [126]. However, over 30% of ACPA- and RF-positive individuals with arthralgia do not develop RA within 2 years of follow-up [125], with the level of ACPA seeming to be of importance. In a recent study, 46% of at-risk persons with high ACPA levels developed RA in a 5 year-period, compared to only 21.5% among all ACPA-positive individuals [127]. Similarly, in another study, 32% of ACPA-positive first-degree relatives of RA patients with the values ≥ 2 cutoffs developed RA in a 5-year period, compared to 26% if the value was just above the cutoff [128].

The positive predictive value (PPV) of ACPA tests, which demonstrates the likelihood of an individual with a positive test to have or develop a condition, varies in different studied populations – from 9% in the general population to 55% when both environmental and genetic risk factors are present, 52% in clinically suspect arthralgia (CSA) and >80% in undifferentiated arthritis patients [129]. Multivariable analysis in one study showed ACPA-positivity to be an independent risk factor for RA development in individuals with CSA (hazard ratio (HR) = 5.1; 2.0–13.2) and to have a 50–67% PPV (RF-negative/RF-positive) for RA development within 2 years [125]*.* Anti-CarP-antibodies also showed an increased HR for RA of 3.9 (1.9–7.7) in univariable analysis [125]. PPVs vary between different studies due to the ambiguous criteria for CSA, the length of the observational follow-up period they have been calculated for, and other potential confounding factors. However, it can be concluded that ACPA and other autoantibodies mostly precede the arthritis phase and have quite a high predictive value in individuals with joint complaints.

During the timeline before the onset of arthritis, such events as epitope spreading [25, 124], Fc-glycosylation changes of IgG ACPA towards a more pro-inflammatory phenotype [130] and expansion of ACPA immunoglobulin isotype repertoire [131] are taking place.

ACPA and RF are included in the EULAR/ACR 2010 classification criteria for RA due to their specificity and high prevalence in patients. On the other hand, there is no advice for using autoantibody monitoring during treatment since their levels usually do not correlate with disease activity. Nevertheless, studies suggest that the presence of multiple AMPA and/or autoantibody isotypes is associated with significantly lower success rates of achieving drug-free remission [132, 133] but might be associated with better early treatment outcomes [132, 134]. Moreover, seropositivity, especially ACPA-positivity, is associated with a better response to some biological disease-modifying anti-rheumatic drugs: especially to rituximab, but also to abatacept, tocilizumab and tofacitinib but not to TNF-inhibitors [135–138].

Collectively, studies show that seropositivity for various autoantibodies in RA is associated with a more severe disease course, radiographic damage and bone loss, even prior to the disease onset [90, 139, 140]. In a recent observational study, various AMPA (to citrullinated, carbamylated, and acetylated peptides) were shown to contribute to the risk of radiographic progression only if present as a combination but not separately [95]. IgA RF was also found to be associated with erosive disease [140, 141].

About 40–30% of RA patients are still being classified as “seronegative RA.” Interestingly, the incidence of RF-negative RA has risen in Minnesota, USA, in the last decade compared to 1995–2004, while the incidence of ACPA-positive or ACPA-negative RA has not changed [142]. It has been discussed for decades, whether these patients truly represent RA or are being misclassified. As mentioned above in the section “Autoantibodies to native proteins”, novel autoantibodies, as well as fine-specificities are detected in a proportion of seronegative RA patients. For example, ACPA fine-specificities were found in 30% of seronegative RA patients in one study and anti-CarP antibodies—in 16% [9]. Taking into account other antibody classes than IgG and tests other than CCP-2, this group of patients will probably be shrinking further in the upcoming years. Moreover, another study showed that after 15 years of follow-up, 8.8% of patients initially diagnosed with seronegative RA have been re-diagnosed with spondyloarthritis [143]. In another cohort, 3% in a 10-year period were re-classified to various rheumatic conditions: polymyalgia rheumatica, psoriatic arthritis, osteoarthritis, spondyloarthritis and others [144]. This indicates the need for more careful diagnostics and follow-up in patients who are diagnosed with seronegative RA.

## Conclusion

Autoimmunity in RA is the subject of intensive research, but despite that fact, multiple unanswered questions remain. A variety of autoantibodies has been described, though the mechanisms of the breach of tolerance and disease progression are still not completely understood. The role of autoantibodies in the pathogenetic cascade is likely to be significant, although many other factors, such as cells of the myeloid lineage, FLS, cytokines, T- and B- cells are also involved and the placing of autoantibodies among them is not an easy task. The expansion of the knowledge on autoimmunity in RA is crucial for better understanding of its development and for further treatment advancements. Some aspects remain to be investigated, such as the origin, role and interplay of different autoantibody isotypes, the importance of epigenetic changes in the autoantibody production and the physiological role and origins of ACPA under “normal” conditions. Expanding the range of clinically tested autoantibodies in seronegative RA and individuals at-risk is an important point for the future of patient care.
